# Supplementary material for: Stepwise Expansion of Antimicrobial Stewardship Programs and Its Impact on Antibiotic Use and Resistance Rates at a Tertiary Care Hospital in Korea
Source: Microbiol Spectr. 2022 Apr 25;10(3):e00335-22. doi: 10.1128/spectrum.00335-22 (PMC9241935; doi:10.1128/spectrum.00335-22)
Supplement: SUPPLEMENTAL FILE 1 — Supplemental material. Download spectrum.00335-22-s001.pdf, PDF file, 0.3 MB [file spectrum.00335-22-s001.pdf]

Supplementary Table 1. Comparison between antibiotic use (days of therapy per 1000 patient-days) at Seoul National University Bundang Hospital and in National Health Insurance Service data[a] from 2010 to 2019

**Antibiotics**	**Data**	**Year**										**Mann–Kendall test**		**Sen's slope (95% CI)**
		**2010**	**2011**	**2012**	**2013**	**2014**	**2015**	**2016**	**2017**	**2018**	**2019**	**Tau**	***P*[b]**	
**All antibacterial agents**	SNUBH	617.49	618.92	608.28	587.00	596.28	596.27	585.71	591.00	582.34	550.81	-0.78	<0.01	-5.29 (-8.51 to -3.46)
	NHIS	635.44	645.37	623.69	613.47	608.42	636.31	686.99	710.86	777.37	952.02	0.56	0.03	23.87 (0.17 to 42.24)
**Third-generation cephalosporins**	SNUBH	115.04	113.85	93.77	95.60	102.86	99.94	110.17	116.41	115.77	108.86	0.20	0.47	1.20 (-1.19 to 4.03)
	NHIS	191.78	189.06	181.66	172.94	165.78	174.56	186.53	197.91	219.30	273.67	0.33	0.21	4.53 (-3.45 to 11.97)
**ß-lactam/ß-lactamase inhibitors**	SNUBH	62.01	59.46	65.09	63.42	74.77	79.67	72.01	73.25	70.70	70.28	0.33	0.21	1.35 (-0.66 to 2.51)
	NHIS	83.08	80.04	77.51	79.00	81.64	82.56	96.30	94.41	106.35	125.00	0.64	0.01	3.76 (0.93 to 6.18)
**Fluoroquinolones**	SNUBH	65.29	55.60	59.15	57.88	54.81	54.82	52.05	51.55	51.42	46.15	-0.87	<0.01	-1.58 (-2.13 to -0.85)
	NHIS	60.21	59.39	58.58	61.60	67.28	69.05	81.01	84.41	91.16	111.82	0.87	<0.01	5.16 (3.01 to 6.76)
**Glycopeptides**	SNUBH	22.88	24.42	25.79	22.08	23.10	22.20	21.60	20.55	20.65	18.81	-0.69	0.01	-0.56 (-0.87 to -0.28)
	NHIS	5.79	6.67	7.17	7.72	8.48	8.99	9.87	11.55	13.31	17.24	1.00	<0.01	0.88 (0.64 to 1.32)
**Fourth-generation cephalosporins**	SNUBH	2.40	2.50	2.59	2.97	4.09	3.47	2.09	1.56	1.53	1.69	-0.29	0.28	-0.12 (-0.29 to 0.16)
	NHIS	2.55	2.98	2.93	3.27	4.44	4.75	4.77	5.15	5.08	8.23	0.91	<0.01	0.37 (0.23 to 0.63)
**Carbapenems[c]**	SNUBH	21.10	18.09	23.30	19.73	19.64	17.57	19.16	19.56	22.70	20.42	-0.02	1.00	-0.03 (-0.41 to 0.59)
	NHIS	5.33	6.32	7.03	8.31	9.56	10.10	11.32	12.98	14.50	18.01	1.00	<0.01	1.17 (1.00 to 1.46)
**Group 1**	SNUBH	8.97	8.39	11.43	9.85	9.45	9.01	10.61	12.29	14.75	12.91	0.60	0.02	0.51 (0.16 to 0.95)
	NHIS	0.22	0.31	0.47	0.59	0.80	1.02	1.24	1.67	1.98	3.15	1.00	<0.01	0.23 (0.17 to 0.33)
**Group 2**	SNUBH	12.13	9.70	11.87	9.88	10.19	8.56	8.55	7.27	7.95	7.51	-0.73	<0.01	-0.51 (-0.71 to -0.26)
	NHIS	5.11	6.01	6.56	7.72	8.77	9.08	10.08	11.31	12.52	14.86	1.00	<0.01	0.93 (0.84 to 1.15)

CI: confidence interval; SNUBH: Seoul National University Bundang Hospital; NHIS: National Health Insurance Service

[a] The NHIS is the sole health insurance provider in Korea and is responsible for all hospitalization-related costs. We reviewed 40% of all antibiotic-related claim data of patients admitted to Korean tertiary care hospitals.

[b] Trend analyses were performed using nonparametric two-sided correlated seasonal Mann–Kendall tests, with $P < 0.05$ indicating that the trend statistically significantly increased or decreased. The magnitude of change per year was estimated using Sen's method.

[c] Group 1 carbapenem: ertapenem. Group 2 carbapenem: meropenem, imipenem-cilastatin, and doripenem.

Supplementary Table 2. Comparison between antimicrobial resistance rates at Seoul National University Bundang Hospital and in the Korean Antimicrobial Resistance Monitoring System data from 2010 to 2019[a]

Bacteria	Antibacterial agents	Data	Period	Mann–Kendall test		Sen's slope (95% CI)
				Tau	*P*[b]	
S. aureus	Oxacillin	SNUBH	2010–2019	-0.85	<0.01	-1.24 (-1.60 to -0.67)
		KARMS	2010–2016	-0.71	0.04	-2.00 (-3.40 to -0.28)
E. coli[c]	Third-generation cephalosporins	SNUBH	2010–2019	1.00	<0.01	1.90 (1.55 to 2.05)
		KARMS	2010–2016	0.90	<0.01	1.92 (1.40 to 2.90)
	Fluoroquinolones	SNUBH	2010–2019	0.87	<0.01	1.22 (0.90 to 1.50)
		KARMS	2010–2016	0.90	<0.01	1.93 (1.30 to 2.60)
K. pneumoniae[c]	Third-generation cephalosporins	SNUBH	2010–2019	-0.02	1.00	-0.04 (-0.77 to 0.50)
		KARMS	2010–2016	0.43	0.23	0.32 (-0.77 to 1.40)
	Fluoroquinolones	SNUBH	2010–2019	-0.02	1.00	-0.10 (-0.90 to 0.75)
		KARMS	2010–2016	0.71	0.04	1.15 (0.30 to 2.40)
P. aeruginosa	Third-generation cephalosporins	SNUBH	2010–2019	-0.64	0.01	-0.75 (-1.15 to -0.37)
		KARMS	2010–2016	0.05	1.00	0.03 (-1.60 to 0.77)
	Fluoroquinolones	SNUBH	2010–2019	-0.64	0.01	-1.40 (-2.12 to -0.57)
		KARMS	2010–2016	-0.05	1.00	-0.05 (-3.00 to 3.40)
	Carbapenems	SNUBH	2010–2019	-0.39	0.15	-0.37 (-0.85 to 0.10)
		KARMS	2010–2016	0.81	0.02	2.03 (0.60 to 2.67)
A. baumannii	Third-generation cephalosporins	SNUBH	2010–2019	0.33	0.21	1.45 (-0.78 to 3.55)
		KARMS	2010–2016	-0.05	1.00	-0.80 (-4.40 to 4.13)
	Fluoroquinolones	SNUBH	2010–2019	0.38	0.15	0.94 (-0.85 to 2.82)
		KARMS	2010–2016	0.14	0.76	0.90 (-5.15 to 4.80)
	Carbapenems	SNUBH	2010–2019	0.47	0.07	1.60 (-0.06 to 4.58)
		KARMS	2010–2016	0.24	0.55	2.34 (-3.27 to 5.03)

CI: confidence interval; SNUBH: Seoul National University Bundang Hospital; KARMS: Korean Antimicrobial Resistance Monitoring System

[a] Antimicrobial susceptibility was determined using the disk-diffusion method or VITEK 2 (bioMe´rieux, Marcy L'etoil, France). Each isolate was classified as resistant or non-resistant, according to the Clinical and Laboratory Standards Institute criteria. The proportions of

isolates of each bacteria species resistant to the following antibiotics were assessed: oxacillin, third-generation cephalosporins (cefotaxime or ceftazidime), fluoroquinolones (ciprofloxacin), and carbapenems (imipenem). National surveillance data on antibiotic resistance rates from 2010 to 2016 at Korean general hospitals with over 100 beds were obtained from KARMS.

[b] Trend analyses were performed using nonparametric two-sided correlated seasonal Mann–Kendall tests, with $P < 0.05$ indicating that the trend statistically significantly increased or decreased. The magnitude of change per year was estimated using Sen's method.

[c] The proportions of carbapenem-resistant *E. coli* and *K. pneumoniae* at SNUBH remained less than 1.14%.

Supplementary Table 3. Antimicrobial stewardship activities performed for hospitalized patients at Seoul National University Bundang Hospital until 2020

ASP core elements	Examples	Initiation
**Hospital leadership**	**Staff** involved in ASP activities (on a part-time basis)[a] at a hospital with over 1300 beds	
	• Two ID physicians (1 internist and 1 pediatrician)	May 2003
	• Three ID physicians (2 internists and 1 pediatrician)	March 2009
	• Four ID physicians (3 internists and 1 pediatrician)	March 2011
	• Five ID physicians (4 internists and 1 pediatrician)	March 2020
	**CDSS launched**	September 2003<br>Last updated in 2017
	**Pharmacy & therapeutics committee**	
	• Establishment of a subcommittee for antibiotics	September 2003
	• Promotion to ASP committee	November 2018
	• Establishment of a new subcommittee for therapeutic drug monitoring of antibiotics	September 2019
**Accountability**	• Creation of an ASP team consisting of ID specialists, pharmacists, and microbiology laboratory staff	November 2018
	• Regular handshake stewardship for the hematology unit	March 2006
**Pharmacy expertise**	• ID training for the pharmacists	March 2013
	• Designation of one full-time ID pharmacist for ASP activities	May 2019
**Action**	**Preauthorization**	
	• Restricted antibiotic approval only, such as broad-spectrum antibiotics (e.g. carbapenems, glycopeptides, polymyxins, and fourth-generation cephalosporins), antifungals (e.g. newer azoles, liposomal amphotericin-B, and echinocandins), and antivirals (e.g. ganciclovir)	May 2003
	• Post-prescription review and feedback through automatic consultation of ID physicians. If the prescription was approved by an ID physician, antibiotics could be administered for 7 days, and in the absence of a decision, antibiotics could be prescribed for 3 days for urgent use. If the ASP team determined that the antibiotic use was inappropriate, they recommended an alternative treatment in their consultation note, and the pharmacy did not prepare the prescribed antibiotics.	August 2011

	**Prospective audit and feedback**	
	• Electronic alerts with automatic consultation of ID physicians for patients with positive blood cultures	August 2011
	• Prevention of redundant combinations of metronidazole or clindamycin with other anti-anaerobic antibiotics	July 2013
	• Prospective review of nine antibiotics: azithromycin, cefoxitin, clindamycin, colistimethate, sulfamethoxazole/trimethoprim, anidulafungin, fluconazole, voriconazole, and acyclovir	July 2014
	• Intravenous to oral conversion of administration of fluoroquinolone and metronidazole	August 2015
	- expanded to six more antibiotics	August 2019
	• "Shorter is better" campaign targeting antibiotics prescribed for more than 2 weeks. All in-hospital long-term antibiotic prescriptions were monitored daily, and the ID pharmacist advised the physicians to discontinue administration in cases where it was plausible.	August 2018
	**Facility-specific treatment guidelines**	
	• Shortening of duration of antibiotic administration for surgical antibiotic prophylaxis via the clinical pathway	
	- less than 5 days	June 2003
	- less than 3 days	November 2009
	- less than 2 days	April 2015
	- less than 24 hours	April 2020
	• Biannual updating of facility-specific guidelines for solid organ transplantation in collaboration with the transplantation teams	April 2009<br>Last updated in May 2019
	**Pharmacologic intervention**	
	• Vancomycin loading using the computerized CDSS	July 2016
	• Renal dosing guidance	
	- computerized dosing recommendations	April 2006
	- dosing pamphlets released to the prescribers	July 2020
	• Daily alerts, particularly for the pharmacists, using an ASP review sheet in the electronic medical record	November 2016
	- intravenous to oral conversion	
	- inappropriate dosing according to indications and renal function	
	- drug interactions and adverse events	

	**Rapid diagnostics**	
	• Multiplex polymerase chain reaction performed in patients with bacteremia with clusters of gram-positive cocci	February 2012
**Tracking**	**Antibiotic use measures**	
	• Monitoring surgical antibiotic prophylaxis	October 2007
	• Monitoring antibiotic administration data from the clinical database (defined daily dose, day of therapy, and length of therapy per 1000 patient-days)	July 2014
	• Hospital-wide point prevalence survey on the appropriateness of antibiotic prescription	August 2018
	**Outcome measures**	
	• Biweekly meetings with the staff at the microbiology laboratory and infection control office	May 2003
	- changed to weekly meetings	March 2017
	• Daily morning conference with the pharmacists	March 2013
**Reporting**	• Regular report on antibiotic resistance rates for important clinical isolates by newsletter	December 2004
	• Regular report on antibiotic use	April 2009
	• Regular report on the proportion of clinical consultations for therapeutic drug monitoring of antibiotics administered for over 7 days	December 2019
**Education**	• Education programs for physicians and pharmacists about antibiotic choice, dosage, and treatment duration for common infections such as pneumonia, urinary tract infection, and skin and soft tissue infection	March 2016
	• Educational materials developed by elective course internal medicine residents and shared with other physicians	March 2016
	• Annual ASP symposium for health-system pharmacists	October 2019

ASP: antimicrobial stewardship program; ID: infectious disease; CDSS: Clinical decision support system

[a] Part-time basis is a concept as opposed to full-time equivalents, and full-time equivalents are defined as working 52 hours per week for ASP-related activities according to the labor laws in Korea.
